# The efficacy and safety of Xuebijing injection for corona virus disease 2019: A protocol for a systematic review and meta-analysis

**DOI:** 10.1097/MD.0000000000023401

**Published:** 2020-12-04

**Authors:** Jiaming Fu, Lili Wu, Yingying Ma, Qun Liang

**Affiliations:** a Heilongjiang University of Traditional Chinese Medicine; b First Affiliated Hospital of Heilongjiang University of Traditional Chinese Medicine, Heilongjiang Province, China.

**Keywords:** corona virus disease 2019, systematic review, xuebijing injection

## Abstract

**Background:**

Corona virus disease 2019 (COVID-19) is an epidemic respiratory infectious disease caused by Severe Acute Respiratory Syndrome Coronavirus 2 infection. Now it is popular all over the world on a large scale. COVID-19 has the characteristics of rapid transmission, atypical clinical symptoms, easy missed diagnosis and misdiagnosis, and so on. which has seriously affected social and economic development and people's health. Severe acute respiratory syndrome corona virus type 2 infection may lead to systemic cytokine storm, which leads to a sharp deterioration of the condition of ordinary patients. At present, no specific drug has been found in the clinical treatment of covid-19, while Xuebijing injection has been widely used in severe patients in China as a traditional Chinese medicine. The aim of this study is to assess the effificacy and safety of Xuebijing injection for COVID-19.

**Methods:**

Before the research, we conducted a comprehensive search on relevant websites. Two professional researchers will gradually screen, read the title, abstract and full text if necessary, and independently select qualified documents according to the inclusion and exclusion criteria. We will conduct a meta-analysis of the results related to COVID-19 to assess the risks of bias and data extraction. The heterogeneity of data will be studied by Cochrane *X*^2^ and *I*^2^ tests. The evaluation of publication bias will be carried out by funnel chart analysis and Eger test.

**Results:**

This review will be disseminated in print by peer-review.

**Conclusion:**

Our research is to scientifically analyze the clinical evidence of Xuebijing injection in treating severe COVID-19 patients.

## 1 Introduction

In December 2019, the first case of infectious pneumonia was reported in Wuhan, China. With the increasing number of infected people, the epidemic spread to the whole world. The World Health Organization announced that the epidemic constituted a public health emergency of international concern and the disease caused by this virus was officially named corona virus disease 2019 (COVID-19).^[1]^ As of September 22 2020, the world has reported 31,400,432 confirmed cases.^[2,3]^

Most severe cases of COVID-19 have difficulty breathing after 1 week, and the severe cases can rapidly progress to acute respiratory distress syndrome (ARDS), metabolic acidosis which is difficult to correct, coagulation dysfunction, septic shock, multiple organ dysfunction syndrome (MODS), and so on.^[4–7]^ At present, there is no specific treatment drug.^[8,9]^

Led by Academician Zhong Nanshan in China, nearly 30 hospitals participated in the clinical randomized controlled trial of “Prospective cohort study on the efficacy of Xuebijing injection in the treatment of pneumonia infected by novel coronavirus (ChiCTR 2000029381),” which proved the therapeutic effect of Xuebijing on severe and critical patients.^[10,11]^ The effective conclusion has been initially confirmed. Xuebijing injection, as a representative drug for the treatment of severe diseases, has been recognized and recommended by the majority of respiratory and critical medicine experts of Chinese and Western medicine, and has been included in the “Diagnosis and Treatment Plan for Pneumonia Infected by novel coronavirus (Trial 7th Edition)” jointly issued by the National Health and Health Commission and state administration of traditional chinese medicine, and has been included in the recommended drugs for severe and critical cases respectively.^[12–16]^ The reason why Xuebijing injection is widely valued by Chinese and Western medicine is not only because of its outstanding clinical curative effect, but also because of its solid evidence base, and it can continuously deepen its research based on clinical needs.^[17–19]^

## 2 Methods and analysis

### 2.1 Study registration

This study has been registered in advance on the website of International prospective register of systematic reviews (PROSPERO, https://www.crd.york.ac.uk/prospero/) with a registration number of CRD42020206366. In addition, this systematic review protocol is reported in accordance with Cochrane reporting expectations, as recommended by the Cochrane handbook.

### 2.2 Inclusion and exclusion criteria

#### 2.2.1 Study design

In this study, the literature was selected as not requiring randomness. Randomized controlled trial is the gold standard for evaluating intervention measures, but the clinical requirements are strict. Non-randomized studies have low evidence-based medicine certificates, but clinical studies are broader. Since COVID-19 started to explode on a large scale at the beginning of this year. There are relatively few clinical observations that are completely consistent with randomized control, it is appropriate to include non-randomized studies in this systematic review and meta-analysis.

#### 2.2.2 Participants

Participants with laboratory-confifirmed COVID-19 will be included in this study, Symptoms include In the case of dyspnea, acute respiratory distress syndrome (ARDS), intractable metabolic acidosis, coagulation dysfunction, septic shock, multiple organ dysfunction syndrome (MODS), and so on. The assay was primarily RC-qPCR confirmed Severe or critically ill patients, and there were no restrictions on the age, sex of the participants.

#### 2.2.3 Intervention

Studies using Xuebijing injection will be included. The intervention dose was 50 mL and the application method was intravenous drip. In addition, the control group did not use other traditional Chinese medicine related preparations.

#### 2.2.4 Outcomes

Primary outcomes: Total clinical effective rate, effective rate of clinical symptoms, disappearance rate of clinical symptoms, treatment time. Secondary outcomes: improvement rate of lung CT, Liver function, kidney function, adverse events.

### 2.3 Study search.

We will search the following sources for the identifification of trials: three English database including PubMed, EMBASE, Cochrane Library Central Register of Controlled Trials and 4 Chinese databases including China National Knowledge Infrastructure database, Wanfang Data Knowledge Service Platform, the VIP information resource integration service platform, China Biology Medicine Disc All the above databases will be searched from the available date of inception until the latest issue (August 2020). No language or publication restriction will be used.

The search strategy of combining subject words with free words will be adopted. Boolean operators will be used to concatenate search terms. The search strategy of PubMed is presented in Table 1.

**Table 1:** Example of PubMed search strategy.

Number	Search terms
1	Mesh descriptor: (Xuebijing Injection) explode all trees
2	((((((Xue-Bi-Jin [Title/Abstract]) OR Xue-Bi-Jin Injection[Title/Abstract]) OR XueBiJin [Title/Abstract]) OR xuebijing [Title/ Abstract]) OR Xuebijing Injection [Title/Abstract])
3	Or 1–2
4	Mesh descriptor: (COVID-19) explode all trees
5	((((((2019 novel coronavirus infection[Title/Abstract] OR 2019-nCoV infection[Title/Abstract]) OR COVID-19 pandemic[Title/Abstract]) OR coronavirus disease-19 [Title/Abstract]) OR 2019-nCoV disease[Title/Abstract]) OR COVID19[Title/Abstract]) OR 2019 novel coronavirus disease[Title/Abstract]) OR coronavirus disease 2019[Title/Abstract]
6	Or 4–5
7	3 and 6

COVID-19 = corona virus disease 2019.

### 2.4 Study selection

**Figure 1:**
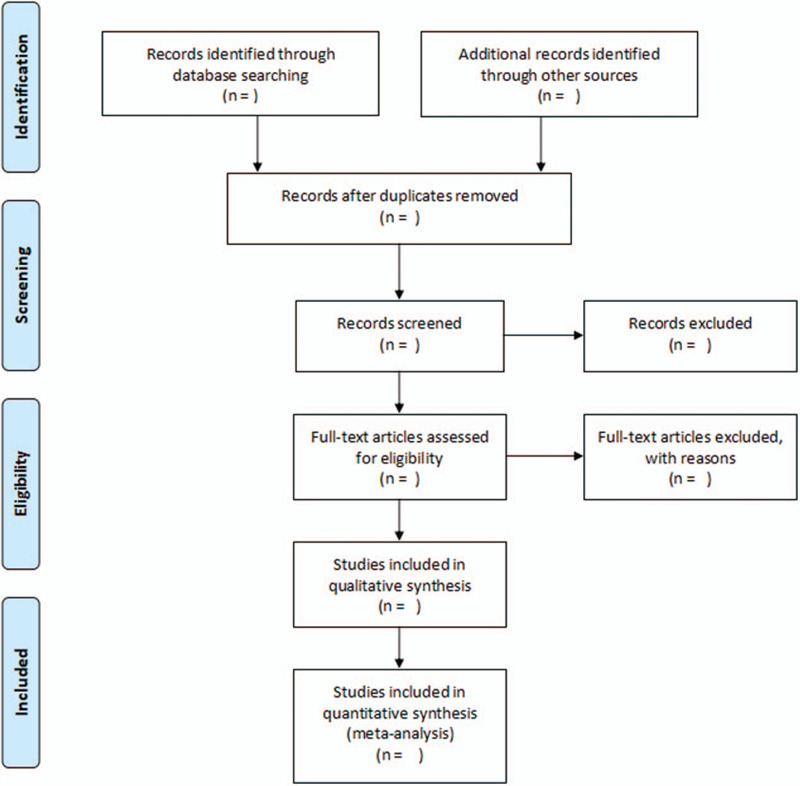
Flow chart of study selection.

EndNote X9 will be used to screen the citations independently according to inclusion and exclusion criteria by 2 reviewers. The differences between the 2 authors will be resolved through discussions with the third author. A research flow chart will be drawn to show the whole process of research selection (Fig. 1).

### 2.5 Data collection and analysis

Data extraction will be conducted by 2 independent authors according to a prespecified form and checked by a third author. The following data will be extracted: the first author's name, publication time, country, article title, article type, interventions in experimental and control group, course of treatment, severity of disease, number of patients in each group, ages and sex of patients, outcomes, Liver function, kidney function and adverse effect. If the author does not report certain information in the article, we will then contact the authors by email for more detailed information. Once the extraction is complete, the 2 authors will check with each other to ensure the accuracy of the data.

### 2.6 Risk of bias assessment

Risk of bias will be independently assessed by 2 authors using the Cochrane tool of risk of bias. Version 2 of the Cochrane risk-of-bias tool for randomized trials and interventions tool for non-randomized studies.

### 2.7 Data analysis

Data analysis will be conducted using Stata 14.0 and RevMan 5.3 software. Continuous variables will be reported as mean difference with 95% confidence intervals (CIs). For different measurement scales, standardized mean difference analysis with 95% CI will be used. Categorical variables will be summarized as risk ratios or odds ratio with 95% CIs. All analysis will be performed based on the continuous variables will be reported as the mean difference of the 95% CIs. Standardized mean difference analysis with 95% confidence interval will be used for different measurement scales. The categorical variables will be summarized as risk ratio or odds ratio and 95% CIs. All analyses will be conducted in accordance with the Cochrane Handbook for Systematic Reviews of Interventions. Heterogeneity will be identifified by visual inspection of the forest and tested by standard Chi-squared statistic and a signifificance level of 0.1. In addition, the *I*^2^ statistic will be used to test heterogeneity to quantify inconsistency. Fixed or random effects models will be performed in meta-analysis. If *I*^2^ > 0.5, the random effects models will be used.

If the information is insufficient or missing, we will contact the study author. If we fail to obtain sufficient data, we will assume dichotomous outcomes for patients not experiencing any change in their clinical outcome variables. Sensitivity analyses will be performed to assess how sensitive the results are to changes in the assumptions made.

## 3 Discussion

Serious cases infected with covid-19 mostly occurred in patients with low immunity and chronic basic diseases, while the treatment cost of severe patients was high and the prevention and treatment cost was poor, which obviously increased the difficulty of prevention and treatment.^[20–23]^ How to effectively prevent covid-19 from progressing from ordinary type to severe type and the prevention and treatment of severe patients deserves further study.^[24]^ The treatment of covid-19 needs to control the increase of lung lesions, inhibit the aggravation of inflammatory reaction and avoid the deterioration of disease; Although a variety of antiviral drugs (including Abidol, Chloroquine, Redcevir, and so on.) and plasma of recovered patients are used in clinical treatment, they have limited effect on blocking the progress of patients.^[25,26]^ This article summarizes the therapeutic effect of Xuebijing injection by collecting evidence systematically, and provides further guidance and promotion for clinical application.

## Author contributions

The protocol was designed by MY, WL and My under the guidance of LQ. All the authors participated in the study. The manuscript was drafted by MY and revised by LQ. All authors approved the fifinal manuscript before submission. MY, WL and My contributed equally to this work and should be regarded as co-fifirst authors.

**Conceptualization:** Jiaming Fu, Qun Liang.

**Data curation:** Jiaming Fu, Lili Wu, Yingying Ma.

**Formal analysis:** Yingying Ma, Lili Wu.

**Investigation:** Jiaming Fu, Lili Wu.

**Methodology:** Jiaming Fu, Lili Wu.

**Project administration**: Qun Liang

**Software:** Jiaming Fu, Lili Wu, Yingying Ma.

**Visualization:** Jiaming Fu, Lili Wu.

**Writing – original draft**: Jiaming Fu, Yingying Ma.

**Writing – review & editing**: Qun Liang.
